# Hepatic‐Differentiated Subpopulation in Clear Cell Renal Cell Carcinoma: A Multi‐Omics Analysis of Tumors With Lymphovascular Invasion

**DOI:** 10.1002/cam4.71843

**Published:** 2026-04-14

**Authors:** Shugo Yajima, Yuichiro Tsukada, Riu Yamashita, Takao Fujisawa, Takeshi Kuwata, Reiko Watanabe, Genichiro Ishii, Nina Gabelia, Hartmut Juhl, Yoshikatsu Koga, Takayuki Yoshino, Masaaki Ito, Hitoshi Masuda

**Affiliations:** ^1^ Department of Urology National Cancer Center Hospital East Kashiwa Japan; ^2^ Department of Colorectal Surgery National Cancer Center Hospital East Kashiwa Japan; ^3^ Division of Translational Informatics Exploratory Oncology Research and Clinical Trial Center, National Cancer Center Kashiwa Japan; ^4^ Translational Research Support Section National Cancer Center Hospital East Kashiwa Japan; ^5^ Department of Genetic Medicine and Services National Cancer Center Hospital East Kashiwa Japan; ^6^ Department of Pathology and Clinical Laboratories National Cancer Center Hospital East Kashiwa Japan; ^7^ Indivumed GmbH Hamburg Germany; ^8^ Translational Research Sample Management Section National Cancer Center Hospital East Kashiwa Japan; ^9^ Department of Gastroenterology and Gastrointestinal Oncology National Cancer Center Hospital East Kashiwa Japan

**Keywords:** clear cell renal cell carcinoma, lymphovascular invasion, metabolic reprogramming, single‐cell RNA sequencing, spatial transcriptomics

## Abstract

The TITANIA (mulTi omIcs daTa cANcer dIagnostics therApies) study is an international collaboration to generate high‐quality multi‐omics cancer profiles. We conducted an exploratory investigation within this framework to understand the molecular basis of lymphovascular invasion (LVI), a critical determinant of metastatic potential in clear cell renal cell carcinoma (ccRCC). We analyzed 31 ccRCC specimens (11 LVI+, 20 LVI−) from the National Cancer Center Hospital East using whole‐genome sequencing, RNA sequencing, and proteomic profiling. Our findings were integrated with public single‐cell RNA sequencing (GSE159115) and spatial transcriptomics (GSE175540) datasets to provide a broader biological context. LVI+ tumors consistently showed a distinctive hepatic‐lineage gene expression signature, with significant upregulation of aldolase B (ALDOB) and other liver‐specific metabolism genes at both the RNA and protein levels. Single‐cell analysis identified a previously unrecognized hepatic‐differentiated tumor subpopulation expressing master transcription factors HNF1A and HNF4A, which was positioned at the terminal stages of tumor evolution. Comparison with established hepatocyte gene signatures from three independent databases confirmed enrichment of hepatic metabolic programs in this subpopulation. Spatial transcriptomics revealed preferential localization within hypoxic tumor regions. A metabolic program resembling hepatic lineage differentiation, associated with aggressive disease features including LVI and hypoxic microenvironments, offers preliminary insights into renal cancer progression and potential biomarker development. This is a discovery‐based, hypothesis‐generating study; all findings require independent functional validation before clinical application.

## Graphical abstract

A hepatic‐differentiated subpopulation is associated with lymphovascular invasion in clear cell renal cell carcinoma. A visual summary of the key findings from our multi‐omics analysis. The TITANIA framework enabled the identification of a hepatic‐differentiated ccRCC subpopulation characterized by the upregulation of liver‐like metabolic pathways and key transcription factors such as HNF1A and HNF4A. This subpopulation expresses a hepatic‐differentiated gene signature that is enriched in LVI+ tumors and predominantly localized in hypoxic tumor regions, suggesting a potential association between metabolic reprogramming, the tumor microenvironment, and lymphovascular invasion. ALB = albumin; ALDOB = aldolase B; ccRCC = clear cell renal cell carcinoma; CTXN3 = cortexin 3; GSEA = gene set enrichment analysis; H‐RCC = hepatic‐differentiated renal cell carcinoma; HE = hematoxylin and eosin; HNF1A = hepatocyte nuclear factor 1A; HNF4A = hepatocyte nuclear factor 4A.

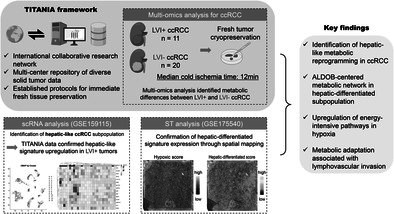

**Abbreviations**
AGXT2: alanine‐glyoxylate aminotransferase 2
ALB: albumin
ALDOB: aldolase B
AMP: amplification
ANGPTL3: angiopoietin‐like 3
ccRCC: clear cell renal cell carcinoma
CIN‐FGA: chromosomal instability‐fraction of genome altered
CNV: copy number variation
DEG: differentially expressed gene
DEL: deletion
DIO1: iodothyronine deiodinase 1
DNA: deoxyribonucleic acid
GSEA: gene set enrichment analysis
HE: hematoxylin and eosin
HNF1A: hepatocyte nuclear factor 1A
HNF4A: hepatocyte nuclear factor 4A
HPD: 4‐hydroxyphenylpyruvate dioxygenase
IQR: interquartile range
KEGG: Kyoto Encyclopedia of Genes and Genomes
LOWCOV: low coverage
LVI: lymphovascular invasion
MSigDB: Molecular Signatures Database
NC: not called
NCCHE: National Cancer Center Hospital East
NES: normalized enrichment score
NEU: neutral
PPI: protein–protein interaction
PRODH: proline dehydrogenase
PROX1: prospero homeobox 1
PT: proximal tubule
RCC: renal cell carcinoma
RIN: RNA integrity number
RNA: ribonucleic acid
scRNA‐seq: single‐cell RNA sequencing
STROBE: Strengthening the Reporting of Observational Studies in Epidemiology
TITANIA: mulTi omIcs daTa cANcer dIagnostics therApies Study
TPM: transcripts per million
UMAP: uniform manifold approximation and projection

## 1 Introduction

Clear cell renal cell carcinoma (ccRCC) is the most common subtype of kidney cancer, characterized by diverse molecular profiles and clinical behaviors [1]. Lymphovascular invasion (LVI), defined as the presence of tumor cells within lymphatic or vascular spaces, is recognized as a significant prognostic factor in ccRCC, associated with increased risk of recurrence and metastasis [2, 3]. Despite its clinical importance, the molecular mechanisms driving LVI in ccRCC remain poorly understood.

The TITANIA (mulTi omIcs daTa cANcer dIagnostics therApies) study is a global collaborative effort to characterize cancer through high‐quality multi‐omics profiling [4], emphasizing rapid freezing of surgical specimens to preserve molecular integrity. As Japan's first participating institution, the National Cancer Center Hospital East (NCCHE) contributed clinical information and samples under stringent quality control. Leveraging this platform, we performed multi‐layered omics profiling of ccRCC specimens, comparing LVI+ and LVI− tumors to identify molecular signatures associated with lymphovascular invasion. We hypothesized that distinct metabolic adaptations could be associated with the propensity of ccRCC cells to invade lymphovascular structures.

## 2 Material and Methods

### 2.1 Patient Cohort and Sample Collection

This study was approved by the National Cancer Center Institutional Review Board (IRB number: 2020‐079) and conducted in accordance with the Declaration of Helsinki. All patients provided written informed consent. The study is reported following the STROBE guidelines.

Thirty‐one consecutive patients with ccRCC who underwent partial or radical nephrectomy at the NCCHE between April 2021 and December 2022 were included. There were no cases of distant metastasis at the time of surgery. Tumor specimens were collected immediately after surgical resection and snap‐frozen in liquid nitrogen, with a median cold ischemia time of 12 min (range 6–26, interquartile range [IQR] 10–13 min). Pathological assessment was performed by board‐certified pathologists according to standard criteria, including determination of LVI status. Based on final pathological examination, 11 cases were classified as LVI+ and 20 as LVI−. All samples underwent pathological quality control requiring tumor content ≥ 30% and necrosis ≤ 30% prior to molecular profiling, in accordance with the TITANIA study protocol.

### 2.2 Data Quality Assessment

To assess the impact of cold ischemia time on molecular data integrity, we examined the correlation between cold ischemia time and proteomic/phosphoproteomic missing value rates across all 31 samples using Spearman rank correlation (Figure S1).

### 2.3 Multi‐Omics Profiling

Deoxyribonucleic Acid (DNA), Ribonucleic Acid (RNA), and protein were extracted simultaneously from the same tissue specimens using the Qiagen AllPrep Universal Kit (Qiagen N.V., Venlo, Netherlands) following the manufacturer's instructions, ensuring consistency across molecular profiling platforms. Approximately 10 mg of fresh‐frozen tissue was used for each extraction, with tissue slices being collected while samples remained frozen to maintain molecular integrity. DNA and RNA quality were assessed using the Agilent Tapestation (Agilent Technologies, Santa Clara, CA, USA) with Genomic DNA and High‐Sensitivity RNA ScreenTape kits, respectively. Only RNA samples with RIN ≥ 4 or DV200 ≥ 60 were selected for sequencing. Whole‐genome sequencing was performed using the PCR‐free KAPA Hyper Prep Kit (Roche Diagnostics, Basel, Switzerland) with an average coverage of ≥ 60× for tumor samples and ≥ 30× for normal samples. RNA sequencing libraries were prepared after ribosomal RNA depletion using the Ribo Zero Kit (Illumina, San Diego, CA, USA) and the TruSeq Stranded Total RNA Kit (Illumina, San Diego, CA, USA), achieving ≥ 100 million total reads with ≥ 20 million reads mapping to mRNAs. Proteomic profiling was conducted using DIA‐NN (v1.8; Biognosys/Evosep) for whole proteome analysis on peptides prepared from 5 to 10 mg tissue, while phosphoproteome analysis was performed using Spectronaut 13 (Biognosys, Schlieren, Switzerland) following Ti‐IMAC magnetic bead enrichment. Batch effects in sequencing data were adjusted using ComBat‐seq. These procedures follow the established protocols in the TITANIA study framework, which emphasizes minimal ischemia time to preserve biomolecular integrity [4]. Detailed sequencing coverage and quality statistics for all samples are provided in Table S1.

### 2.4 Genomic Analysis

All sequencing data were aligned against the GRCh38 reference genome. Somatic variant analysis was performed using a rigorous bioinformatic pipeline that incorporated multiple variant callers including Mutect2, Strelka, VarScan, and SomaticSniper in a consensus approach. Mitochondrial genes were specifically excluded from downstream analyses to focus on nuclear genomic alterations. Copy number variation (CNV) analysis was performed using TitanCNA [5], which jointly models and estimates tumor cellularity (purity) and ploidy as integral parameters of its algorithm. Structural variants were analyzed using DellyCNV and Manta. Chromosomal instability metrics, including fraction of genome altered (CIN‐FGA), were calculated as previously described [6].

### 2.5 Transcriptomic and Proteomic Analysis

RNA‐seq data were processed using standard pipelines for quality control, alignment, and quantification. Differential expression analysis was performed using DESeq2 (v1.44) [7], with significance thresholds set at log_2_ Fold Change (FC) > 1.5 and *p* < 0.05. Proteomic and phosphoproteomic data were processed using DIA‐NN for whole proteome analysis and Spectronaut 13 for phosphoproteome analysis, respectively. For protein expression analysis, missing values were handled using *k*‐nearest neighbor (KNN) imputation [8] to maintain data integrity before differential analysis. Differential protein abundance was determined using limma [9] after imputation, applying the same significance thresholds as for transcriptomic data.

### 2.6 Pathway Enrichment and Network Analysis

Pathway enrichment analysis was performed using clusterProfiler (v4.12) for KEGG pathways [10] and Gene Set Enrichment Analysis (GSEA) for hallmark gene sets [11]. For liver‐specific pathway analysis, a customized approach was implemented by filtering MSigDB gene sets (both C2 and Hallmark collections) [11] using liver‐related keywords such as “LIVER”, “HEPAT”, “BILE”, and “METABOLISM”. Protein–protein interaction (PPI) networks were constructed using the STRING database [12] and visualized with igraph, with hub genes defined as those with at least 8 connections.

### 2.7 Analysis of Public Datasets

We re‐analyzed public single‐cell RNA sequencing (scRNA‐seq) data from GSE159115 [13], specifically focusing on the ccRCC samples. This subset comprised approximately 26,500 cells from 7 ccRCC tumor specimens and 6 matched benign kidney specimens. Data preprocessing and analysis were performed using the Seurat package (v5) [14]. This included quality control filtering (to remove low‐quality cells and genes), data normalization, and identification of highly variable features. Samples from the 13 donors were merged using Seurat's merge() function without explicit inter‐sample batch integration, as all specimens in GSE159115 were derived from a single study using a uniform 10× Chromium protocol [13]. Inter‐sample technical variance was addressed by standard log‐normalization (NormalizeData), selection of 2000 highly variable features using the variance‐stabilizing transformation method, and z‐score scaling prior to dimensionality reduction. The biological validity of the identified clusters was further confirmed by multiple independent downstream analyses (see Comparison with hepatocyte gene signatures below). Cell clustering was conducted using a resolution parameter of 0.1, followed by dimensional reduction and visualization with uniform manifold approximation and projection (UMAP). To trace tumor cell evolutionary trajectories, we performed pseudotime analysis using Monocle3 (v1.3) [15]. Immune cell populations (myeloid cells, T cells, and B cells) were excluded from this analysis, and normal proximal tubule cells were designated as the root state. The GSE159115 dataset was not generated under TITANIA‐standardized ischemia conditions; therefore, integration with NCCHE multi‐omics data may introduce subtle batch‐related biological variance.

Transcription factor (TF) activity was inferred using the VIPER algorithm, leveraging regulons derived from DoRothEA [16]. To investigate the spatial context of gene expression, we analyzed public spatial transcriptomics data from a ccRCC study (GSE175540 [17]), encompassing 24 tumor samples, following established protocols [18]. *K*‐means clustering with three centers was applied to group spatial spots into distinct microenvironmental regions based on expression of RCC markers (CA9, SLC2A1, VEGFA, PAX8) and hypoxia markers (CA9, HIF1A, EPAS1, LDHA, PDK1, SLC2A1). This approach enabled classification of tissue regions as tumor hypoxic, tumor normoxic, or non‐tumor, allowing for spatial correlation analysis of hepatic‐differentiated gene signature expression with hypoxic regions and lymphatic structures (PROX1). Spot‐level expression was normalized using SCTransform (assay = “Spatial”). The hepatic‐differentiated RCC gene signature score was calculated for each spatial spot using Seurat's AddModuleScore function, applying the gene list derived from Cluster 4 (Table S5). No cross‐sample batch correction was applied to the spatial data, as all 24 samples in GSE175540 were derived from a single published study using a uniform 10× Visium platform [17].

### 2.8 Comparison With Hepatocyte Gene Signatures

To evaluate whether the hepatic‐like tumor subpopulation identified in scRNA‐seq analysis (Cluster 4; see Results) represents genuine hepatocyte‐like differentiation, its differentially expressed genes (DEGs) were compared with hepatocyte gene signatures from three independent databases: CellMarker 2.0 (human hepatocyte markers), the Human Protein Atlas (liver‐enriched genes), and MacParland et al. [19] (normal liver scRNA‐seq hepatocyte markers). Overlap significance was assessed by Fisher's exact test (background: 20,000 genes). Module scores were calculated using Seurat's AddModuleScore function [14]. Functional category analysis classified overlapping genes into hepatocyte‐specific pathways to determine whether the overlap reflected broad hepatocyte features or selective acquisition of specific functional programs.

### 2.9 External Validation Using TCGA‐KIRC Cohort

To assess prognostic relevance in an independent cohort, we analyzed TCGA‐KIRC RNA‐seq data [1]. Processed expression data (Illumina HiSeq, RSEM normalized) and clinical information were obtained from the UCSC Xena platform [20]. Survival endpoints were defined per Liu et al. [21]. A weighted signature score was calculated as the mean expression of signature genes weighted by log_2_FC. Among TCGA‐KIRC patients who underwent curative‐intent surgery and for whom disease‐free interval (DFI) data were available, patients were dichotomized using the optimal cutpoint. Kaplan–Meier analysis and log‐rank tests were performed using the survival (v3.5‐7) and survminer (v0.4.9) packages in R.

### 2.10 Statistical Analysis

Statistical analyses were performed using R version 4.4.1. Comparisons between groups were made using Wilcoxon rank‐sum tests for continuous variables and Fisher's exact tests for categorical variables. Spearman rank correlation was used for continuous variable associations. All differential expression analyses applied Benjamini‐Hochberg false discovery rate (FDR) correction; adjusted *p* < 0.05 was used as the significance threshold throughout. For the TCGA‐KIRC survival analysis, the optimal dichotomization cutpoint was determined by maximally selected rank statistics (surv_cutpoint in survminer); this approach may overestimate statistical significance, and results should not be interpreted as confirmatory due to post hoc thresholding.

## 3 Results

### 3.1 Study Design and Clinical Characteristics

**FIGURE 1:**
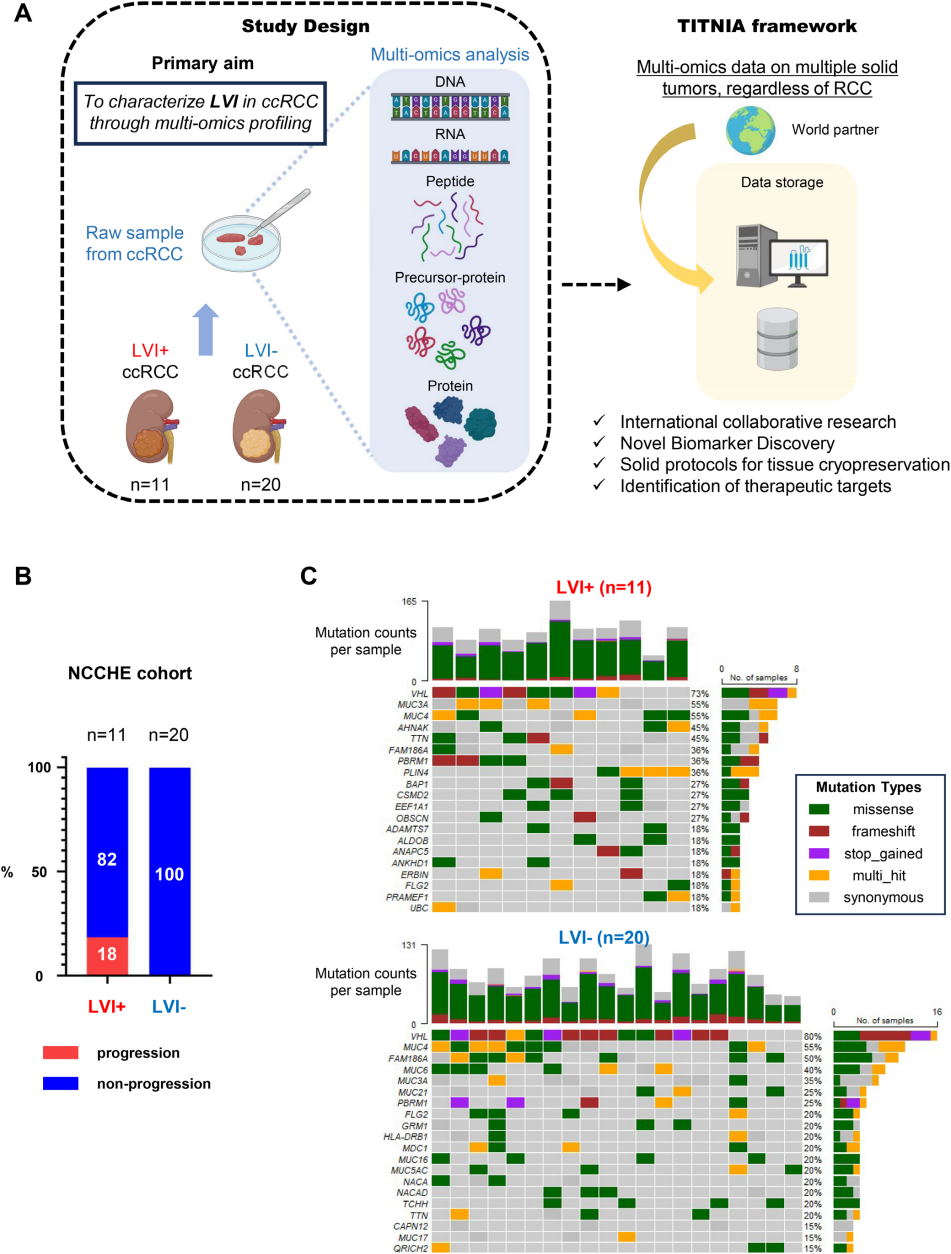
TITANIA multi‐omics investigation: Study design and baseline characteristics of lymphovascular invasion in ccRCC subproject. (A) Schematic illustration of the study design within the broader TITANIA international collaborative project framework. This study aims to characterize lymphovascular invasion (LVI) in clear cell renal cell carcinoma (ccRCC) through comprehensive multi‐omics profiling. Fresh surgical specimens from 31 ccRCC patients (11 LVI+ and 20 LVI−) were subjected to multi‐layered omics analysis including DNA sequencing, RNA transcriptomics, and protein/peptide profiling. The TITANIA project integrates multi‐omics data from multiple solid tumors across global research partners to discover novel biomarkers and potential therapeutic targets. (B) Clinical outcomes in the National Cancer Center Hospital East (NCCHE) cohort, stratified by LVI status. Among LVI+ patients (*n* = 11), 2 (18.2%) patients experienced disease progression during the follow‐up period (median 18.3 months), while all LVI− patients (*n* = 20) remained progression‐free. (C) Oncoplot depicting somatic mutation profiles of ccRCC tumors segregated by LVI status. The upper panel shows mutation patterns in LVI+ samples while the lower panel displays the mutation landscape in LVI− samples. In both groups, VHL mutations occurred with the highest frequency. The top histogram indicates mutation counts per sample, while the right histogram shows the number of samples with mutations in each gene. Different mutation types are color‐coded according to the legend. VHL was the most frequently mutated gene in both groups (LVI+ 73%, LVI− 80%), consistent with established ccRCC genomic profiles. Sample sizes: LVI+ (*n* = 11), LVI− (*n* = 20). ccRCC = clear cell renal cell carcinoma; LVI = lymphovascular invasion; NCCHE = National Cancer Center Hospital East; TITANIA = mulTi omIcs daTa cANcer dIagnostics therApies Study. Sequencing was performed on the Illumina NovaSeq6000 platform using PCR‐free KAPA Hyper Prep Kit library preparation, with all data aligned to the GRCh38 reference genome.

As part of the international TITANIA collaborative project, we conducted comprehensive multi‐omics profiling of 31 ccRCC specimens (11 LVI+ and 20 LVI−) collected at NCCHE (Figure 1A). Patient demographics and tumor characteristics are summarized in Table 1. Patients with LVI+ tumors were significantly older than those with LVI− tumors (median age 79 vs. 63 years, *p* = 0.013). The LVI+ group showed significantly higher T stages and tumor grades compared to the LVI− group (*p* = 0.0039 and *p* = 0.0002, respectively, Table 1). Gender distribution and cold ischemia time were not significantly different between groups. During a median follow‐up of 18.3 months, 2 patients (18.2%) in the LVI+ group experienced disease progression with distant metastasis, while all patients in the LVI− group remained progression‐free (Figure 1B).

**TABLE 1:** Patient characteristics of LVI+ and LVI− groups in ccRCC cohort (NCCHE, *n* = 31).

Characteristic	LVI− (*n* = 20)	LVI+ (*n* = 11)	*p*
Age, years			
Median (IQR)	63 (51.5–73)	79 (73–82)	0.013*
Range	36–88	43–88	
Gender, *n* (%)			0.41
Female	5 (25.0%)	5 (45.5%)	
Male	15 (75.0%)	6 (54.5%)	
T stage, *n* (%)			0.0039*
T1a	8 (40.0%)	0 (0.0%)	
T1b	6 (30.0%)	1 (9.1%)	
T2a	3 (15.0%)	2 (18.2%)	
T3a	3 (15.0%)	7 (63.6%)	
T4	0 (0.0%)	1 (9.1%)	
Grading, *n* (%)			0.0002*
G1	7 (35.0%)	1 (9.1%)	
G2	12 (60.0%)	4 (36.4%)	
G3	0 (0.0%)	5 (45.5%)	
G4	0 (0.0%)	1 (9.1%)	
NA	1 (5.0%)	0 (0.0%)	
Cold ischemia time, minutes			0.1
Median (IQR)	12 (10–13)	13 (9–18)	
Range	6–16	7–26	
Perinephric fat invasion, *n* (%)			0.42
Present	3 (15.0%)	3 (27.3%)	
Absent	17 (85.0%)	8 (72.7%)	

Abbreviations: ccRCC = clear cell renal cell carcinoma, IQR = interquartile range, LVI = lymphovascular invasion, NA = not available, NCCHE = National Cancer Center Hospital East.

* Statistically significant (*p* < 0.05).

### 3.2 Genomic Landscape of LVI+ Versus LVI− ccRCC

**FIGURE 2:**
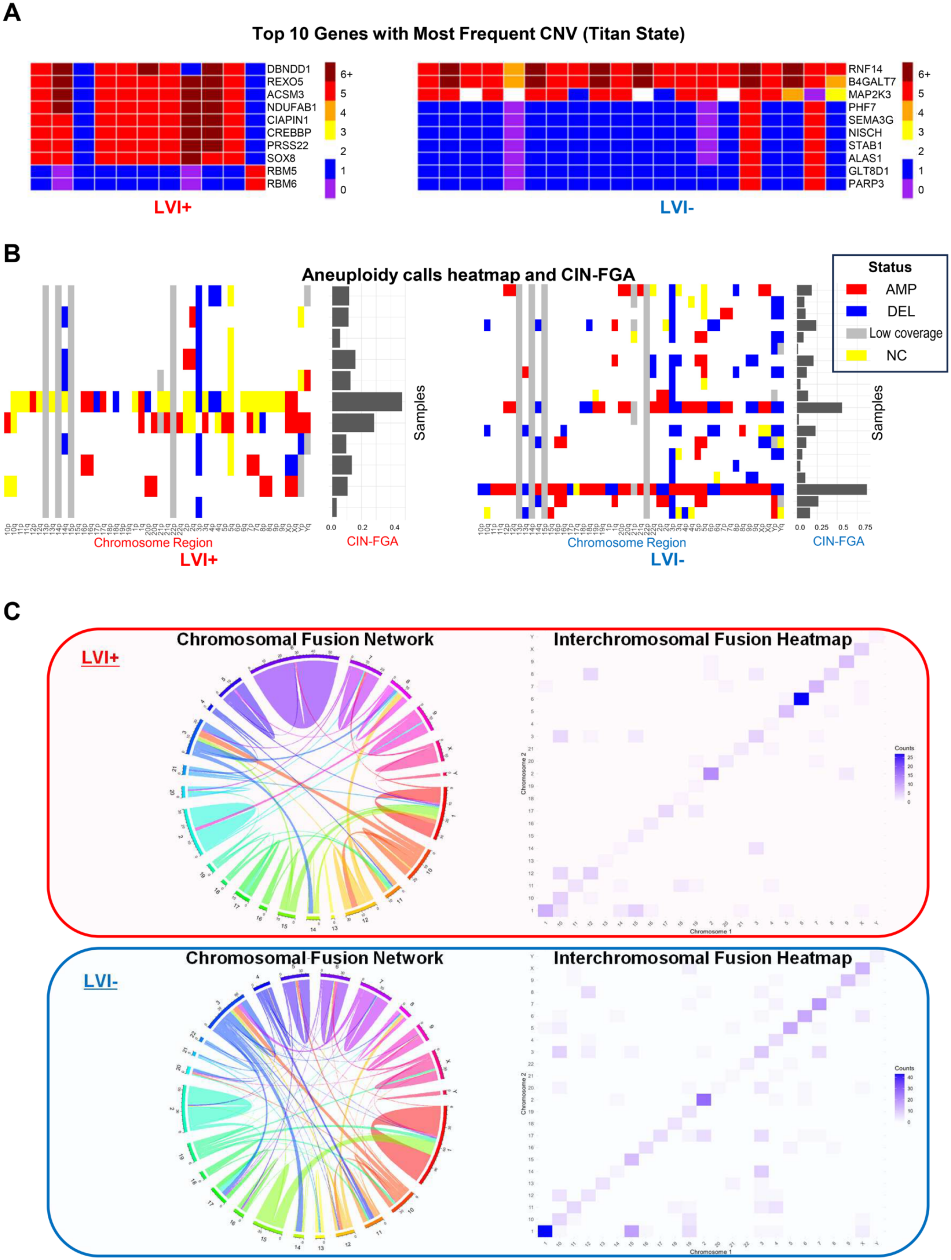
Genomic alterations and chromosomal instability in LVI+ versus LVI− clear cell renal cell carcinoma. (A) Heatmap depicting the top 10 genes with the most frequent copy number variations (CNVs) by Titan state in LVI+ (left) and LVI− (right) cohorts. The color scale represents different Titan states ranging from 0 (homozygous deletion) to 6+ (high‐level amplification). (B) Aneuploidy calls heatmap showing chromosomal alterations across different chromosome regions for LVI+ (left) and LVI− (right) tumors. Red indicates amplifications (AMP), blue represents deletions (DEL), yellow shows not called regions (NC), and gray denotes low coverage regions (low coverage). The corresponding chromosomal instability scores (CIN‐FGA: Fraction of genome altered) are shown as bar graphs on the right side of each heatmap. (C) Chromosomal fusion analysis comparing LVI+ (top panel) and LVI− (bottom panel) cohorts, showing aggregated medium and high confidence fusion events across all samples in each group. Left: Circos plots displaying interchromosomal fusion events, with chromosomes arranged in a circular layout and colored lines representing fusion junctions between different chromosomes. Right: Heatmaps quantifying the frequency of fusion events between chromosome pairs, with darker blue indicating higher numbers of fusion events. AMP = amplification; ccRCC = clear cell renal cell carcinoma; CIN‐FGA = chromosomal instability‐fraction of genome altered; CNV = copy number variation; DEL = deletion; LVI = lymphovascular invasion; NC = not called; NCCHE = National Cancer Center Hospital East; TITANIA = mulTi omIcs daTa cANcer dIagnostics therApies Study.

Having established the clinical framework, we systematically characterized each molecular layer. To investigate the genomic alterations associated with LVI, we performed whole‐genome sequencing of tumor tissue samples. Sequencing coverage details are provided in Table S1. Somatic mutation analysis revealed VHL as the most frequently mutated gene in both groups (LVI+ 73%, LVI− 80%), consistent with established ccRCC genomic profiles [1, 22] (Figure 1C). We next examined copy number variations (CNVs) and chromosomal instability (Figure 2A,B). Both LVI+ and LVI− tumors displayed characteristic deletions on chromosome 3p (containing VHL, PBRM1, and BAP1) and 14q, which are frequently observed in ccRCC [23]. Chromosomal fusion analysis revealed patterns of interchromosomal connections in both LVI+ and LVI− tumors, suggesting some differences in the distribution of fusion events between the two groups (Figure 2C). Statistical comparison revealed significantly higher tumor mutational burden (TMB) in LVI+ tumors (median 3.37 vs. 2.31 mutations/Mb, Wilcoxon *p* = 0.030), while chromosomal instability metrics (CIN‐FGA, CIN‐CNA, CIN‐CNH) did not differ significantly (all *p* > 0.7; Table S2). CNV status of key driver and study‐relevant genes showed no significant differences between groups (Table S3). The higher TMB in LVI+ tumors may partly reflect the significantly older age of these patients, as TMB is known to increase with age, and should be interpreted with caution given the small sample size.

### 3.3 Transcriptomic and Proteomic Profiling Identifies a Metabolic Signature in LVI+ ccRCC

**FIGURE 3:**
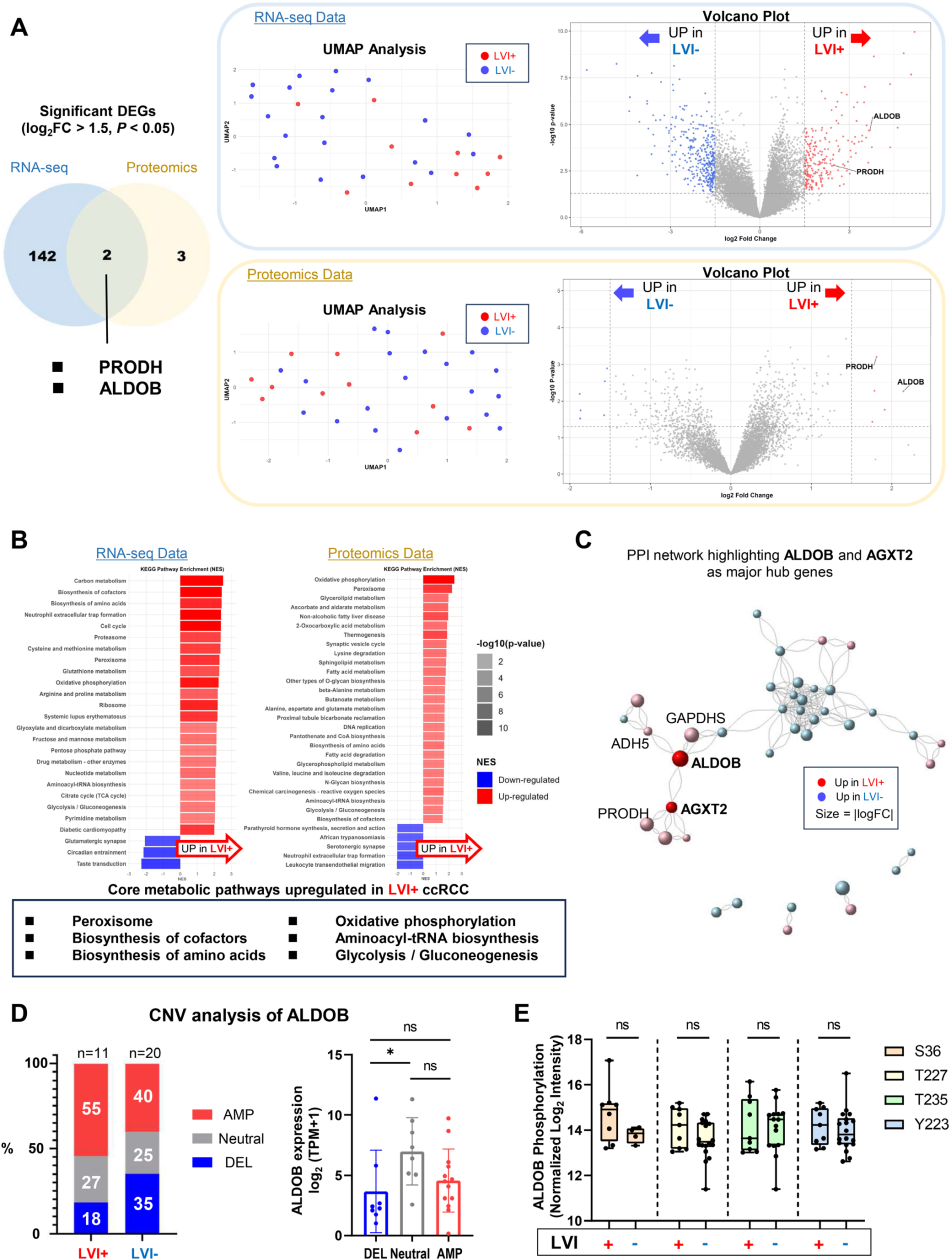
Metabolic alterations in lymphovascular invasion‐positive clear cell renal cell carcinoma. (A) Integrated transcriptomic and proteomic analysis of LVI+ and LVI− tumors. UMAP plots show distinct clustering patterns for both RNA‐seq (top) and proteomic (bottom) data, with LVI+ samples in red and LVI− in blue. Corresponding volcano plots reveal differentially expressed genes and proteins. The Venn diagram highlights ALDOB and PRODH as the only molecules significantly upregulated (log_2_FC > 1.5, *p* < 0.05) in both datasets. (B) KEGG pathway enrichment analysis of differentially expressed genes and proteins in LVI+ versus LVI− tumors. Six core metabolic pathways were commonly upregulated across both modalities, including peroxisome function, biosynthesis of cofactors, biosynthesis of amino acids, oxidative phosphorylation, aminoacyl‐tRNA biosynthesis, and glycolysis/gluconeogenesis. (C) Protein–protein interaction network of differentially expressed proteins. ALDOB and AGXT2 function as major hub genes with multiple interaction partners. Node size represents absolute log fold change magnitude; red indicates upregulation in LVI+ samples, blue indicates downregulation. (D) ALDOB copy number variation analysis comparing LVI+ (*n* = 11) and LVI− (*n* = 20) tumors. Left: Distribution of amplification (AMP), neutral, and deletion (DEL) states. Right: ALDOB expression levels (log_2_TPM + 1) across different CNV states, showing significantly lower expression in DEL compared to neutral samples (*p < 0.05). (E) Analysis of ALDOB phosphorylation at four sites (S36, T227, T235, Y223) shows no significant differences between LVI+ and LVI− tumors, indicating that phosphorylation at these sites may not be the primary regulatory mechanism for ALDOB in this context. AGXT2 = alanine‐glyoxylate aminotransferase 2; ALDOB = aldolase B; AMP = amplification; CNV = copy number variation; DEG = differentially expressed gene; DEL = deletion; KEGG = Kyoto Encyclopedia of Genes and Genomes; LVI = lymphovascular invasion; NES = normalized enrichment score; PPI = protein–protein interaction; PRODH = proline dehydrogenase; TPM = transcripts per million; UMAP = uniform manifold approximation and projection.

We next examined the transcriptomic and proteomic landscape to identify molecular pathways associated with LVI. Unsupervised clustering using UMAP showed trending separation between LVI+ and LVI− samples in both RNA‐seq and proteomic data (Figure 3A). Differential expression analysis identified significantly upregulated genes and proteins (log_2_FC > 1.5, *p* < 0.05) in LVI+ versus LVI− tumors. Notably, aldolase B (ALDOB) and proline dehydrogenase (PRODH) were consistently upregulated at both RNA and protein levels (Figure 3A).

Pathway enrichment analysis of differentially expressed genes and proteins revealed significant upregulation of six core metabolic pathways in LVI+ tumors: peroxisome function, biosynthesis of cofactors, biosynthesis of amino acids, oxidative phosphorylation, aminoacyl‐tRNA biosynthesis, and glycolysis/gluconeogenesis (Figure 3B). These findings suggest that LVI+ tumors undergo substantial metabolic reprogramming, particularly affecting energy production and macromolecule biosynthesis.

PPI network analysis of differentially expressed proteins identified ALDOB and alanine‐glyoxylate aminotransferase 2 (AGXT2) as major hub genes, both involved in metabolic processes (Figure 3C). The prominence of ALDOB, a key enzyme in fructose metabolism primarily expressed in the liver and kidney, prompted us to investigate its potential role in LVI.

Given the significant upregulation of ALDOB in LVI+ tumors, we examined its genomic status and potential regulatory mechanisms. CNV analysis revealed a higher frequency of ALDOB amplifications in LVI+ tumors (54.5% vs. 40.0% in LVI−) and fewer deletions (18.2% vs. 35.0% in LVI−), although neither difference reached statistical significance (*p* = 0.48 for amplifications; *p* = 0.43 for deletions) (Figure 3D). Interestingly, ALDOB RNA expression was significantly lower in samples with ALDOB deletions compared to those with neutral copy number status (*p* < 0.05), potentially indicating a relationship between gene copy number and expression levels. The absence of significant CNV differences despite expression‐level upregulation suggests transcriptional regulation as the primary mechanism, consistent with the activation of HNF1A and HNF4A observed in single‐cell analysis (see below).

To investigate potential post‐translational regulation, we analyzed ALDOB phosphorylation at four sites (S36, T227, T235, and Y223) identified in our phosphoproteomic data. No significant differences in phosphorylation levels were observed between LVI+ and LVI− tumors at any of these sites (Figure 3E), indicating that phosphorylation at these specific residues may not be the primary regulatory mechanism for ALDOB in this context.

### 3.4 Single‐Cell RNA Sequencing Reveals a Hepatic‐Differentiated Subpopulation in ccRCC

**FIGURE 4:**
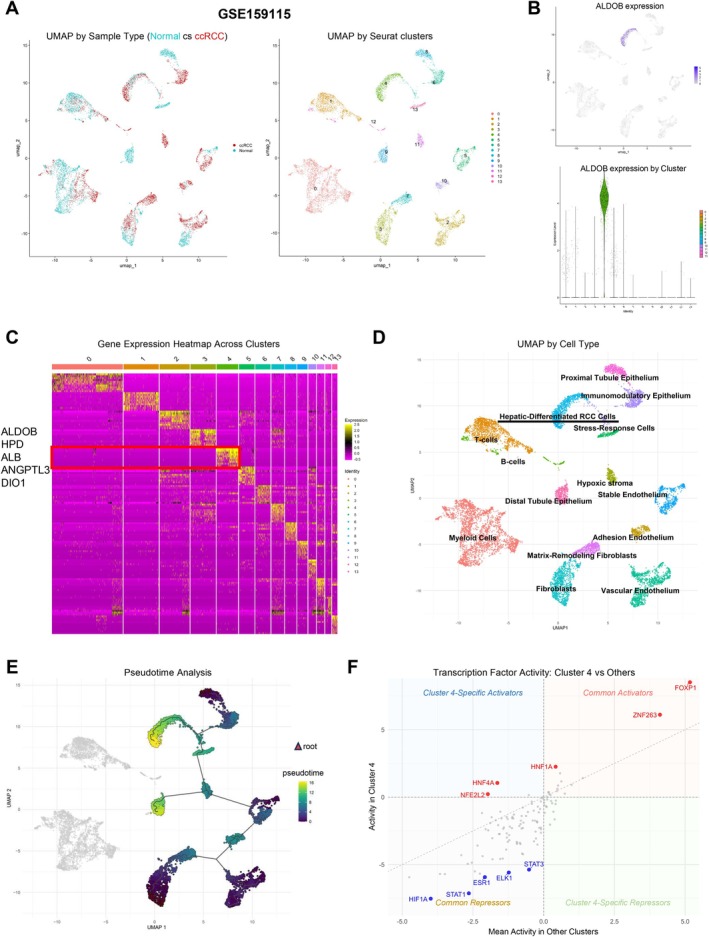
Public data analysis of single‐cell transcriptomic profiles reveals a unique hepatic‐like subpopulation in clear cell renal cell carcinoma. (A) UMAP visualization of scRNA‐seq data from GSE159115. Left panel shows sample distribution with normal kidney tissue (teal) and ccRCC tumor tissue (red). Right panel displays 14 distinct cell clusters identified through unsupervised clustering. (B) ALDOB expression analysis. Upper panel: Feature plot showing ALDOB expression intensity across all cells. Lower panel: Violin plot demonstrating significantly elevated ALDOB expression specifically in cluster 4. (C) Gene expression heatmap across all clusters showing that cluster 4 is characterized by high expression of hepatocyte‐associated genes including ALDOB, HPD, ALB, ANGPTL3, and DIO1, suggesting a hepatic‐like phenotype. (D) Functional annotation of cell clusters based on marker gene expression profiles. Cluster 4 is identified as “Hepatic‐Differentiated RCC Cells,” representing a unique tumor subpopulation with liver‐like features. (E) Pseudotime trajectory analysis using normal proximal tubule cells as root cells, excluding immune cell populations. The color gradient indicates pseudotime progression, with cluster 4 appearing at terminal stages of the differentiation trajectory. (F) Transcription factor activity analysis comparing cluster 4 versus other non‐immune clusters. Top 5 upregulated (red) and downregulated (blue) transcription factors are labeled. Key hepatic lineage transcription factors (HNF1A, HNF4A) show significant upregulation in cluster 4, confirming the hepatic‐like differentiation program. Diagonal dashed line indicates equal activity between cluster 4 and other clusters. ALB = albumin; ALDOB = aldolase B; ANGPTL3 = angiopoietin‐like 3; ccRCC = clear cell renal cell carcinoma; DIO1 = iodothyronine deiodinase 1; HNF1A = hepatocyte nuclear factor 1A; HNF4A = hepatocyte nuclear factor 4A; HPD = 4‐hydroxyphenylpyruvate dioxygenase; PT = proximal tubule; RCC = renal cell carcinoma; scRNA‐seq = single‐cell RNA sequencing; UMAP = uniform manifold approximation and projection.

Building on these bulk transcriptomic and proteomic findings, we used single‐cell resolution to further characterize the ALDOB‐expressing cell population. To this end, we analyzed a public single‐cell RNA sequencing dataset (GSE159115) [13]. UMAP clustering identified 14 distinct cell clusters, representing both normal kidney cell types and malignant cells (Figure 4A).

Analysis of ALDOB expression across all clusters revealed strikingly high expression specifically in cluster 4, a tumor‐derived population (Figure 4B). Further characterization of cluster 4 revealed co‐expression of multiple hepatocyte‐associated genes, including HPD, ALB, ANGPTL3, and DIO1 (Figure 4C). Based on this distinctive gene expression profile, we classified cluster 4 as “Hepatic‐Differentiated RCC Cells,” representing a unique tumor subpopulation with liver‐like features (Figure 4D and Table S4).

Cluster 4 comprised approximately 7.9% of all profiled cells (917/11,662) in GSE159115, representing a distinct but not rare subpopulation. To rigorously assess whether Cluster 4 resembles actual hepatocytes, we compared its DEGs with established hepatocyte gene signatures from CellMarker 2.0, the Human Protein Atlas, and MacParland et al. [19]. Significant overlap was observed with all three databases (Fisher's exact test, *p* < 0.001 for all). Module score analysis confirmed Cluster 4 had the highest hepatocyte scores across all sources (Wilcoxon *p* < 0.001; Figure S2). Functional category analysis (Figure S3) revealed selective acquisition of hepatic metabolic programs—gluconeogenesis/fructose metabolism (3/5 genes, 60%), lipid transport (5/11, 45%), amino acid metabolism (4/10, 40%)—while non‐metabolic hepatocyte functions were absent: coagulation (0/10, 0%), transporters (0/6, 0%), one‐carbon metabolism (0/3, 0%). This pattern indicates selective metabolic adaptation rather than complete hepatocyte trans‐differentiation.

To investigate the developmental trajectory of these hepatic‐differentiated cells, we performed pseudotime analysis using normal proximal tubule cells as the root population. This analysis positioned cluster 4 at the terminal stages of the differentiation trajectory (Figure 4E), suggesting that hepatic‐differentiated cells may represent an evolved state of ccRCC cells that have undergone substantial transcriptional reprogramming.

Transcription factor activity analysis comparing cluster 4 to other non‐immune clusters revealed significant upregulation of key hepatic lineage transcription factors, including hepatocyte nuclear factor 1A (HNF1A) and hepatocyte nuclear factor 4A (HNF4A) (Figure 4F). These master regulators are known to drive hepatocyte differentiation and liver‐specific metabolic programs [24], further supporting the hepatic‐like nature of this ccRCC subpopulation.

### 3.5 Expression of Hepatic‐Differentiated RCC Gene Signature in Relation to Tumor Hypoxia and LVI Status

**FIGURE 5:**
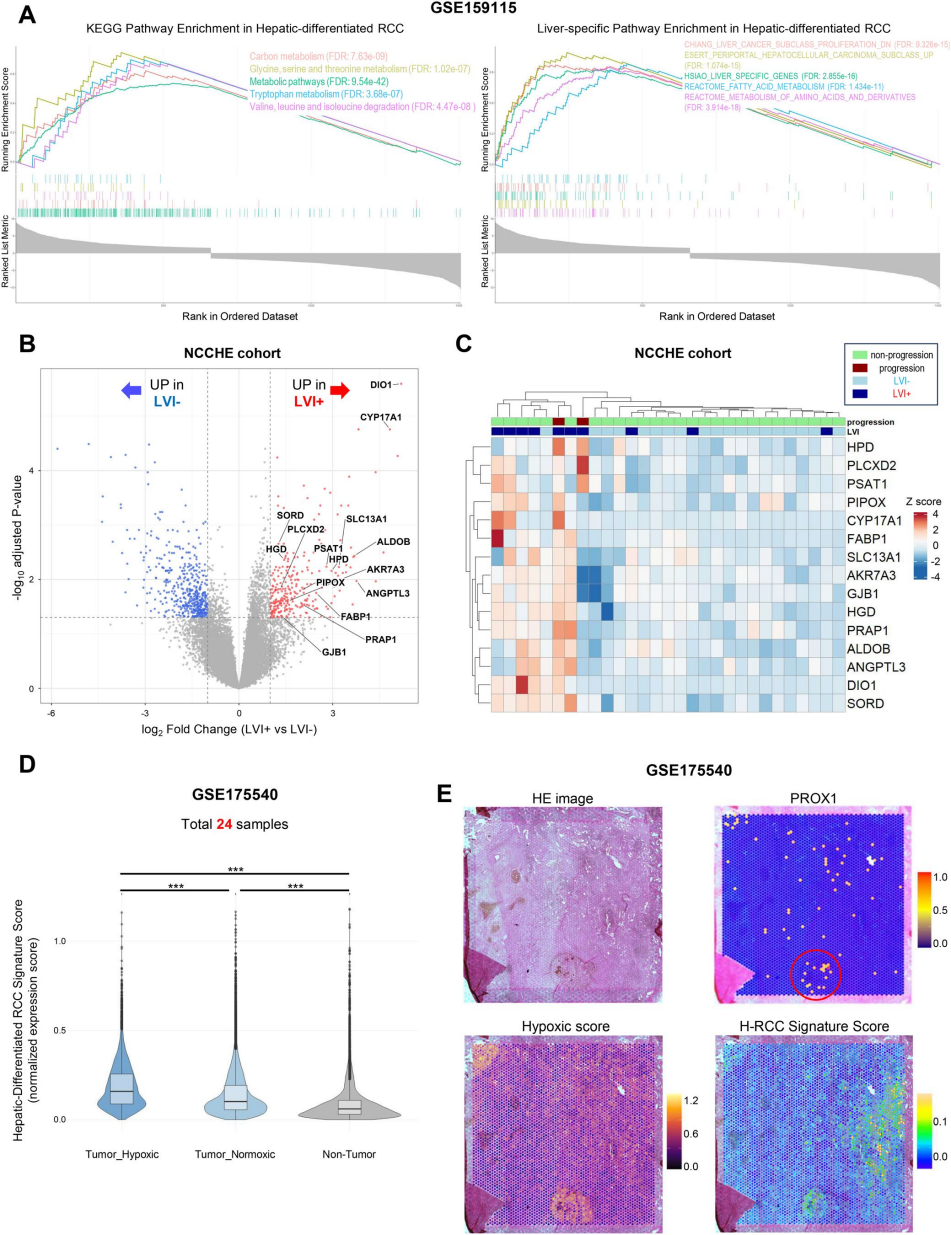
Hepatic‐differentiated signature in clear cell renal cell carcinoma: Association with lymphovascular invasion and hypoxic microenvironment. (A) Pathway enrichment analysis of GSE159115 showing enrichment patterns in cluster 4 (hepatic‐differentiated RCC cells). Left: Top 5 upregulated KEGG pathways with FDR‐adjusted *p*‐values shown in parentheses, including carbon metabolism and amino acid metabolism, revealing enrichment of energy‐related metabolic processes. Right: Significant enrichment of liver‐specific gene programs with FDR‐adjusted *p*‐values. (B) Volcano plot showing differential gene expression in LVI+ versus LVI− tumors from the NCCHE cohort. Liver‐specific metabolic genes (ALDOB, HPD, ANGPTL3, etc.) are among the significantly upregulated genes in LVI+ tumors, suggesting an association between hepatic‐like metabolism and invasive phenotype. (C) Heatmap displaying the expression pattern of hepatic‐differentiated gene signature across the NCCHE cohort, grouped by LVI status and disease progression. The distribution pattern indicates a potential correlation between this gene signature and clinical features. (D) Spatial transcriptomics analysis from GSE175540 showing higher expression of hepatic‐differentiated RCC signature in hypoxic tumor regions compared to normoxic tumor and non‐tumor regions (*p* < 0.001). This spatial distribution suggests an association between hypoxic microenvironments and hepatic‐like gene expression in ccRCC. The asterisks (***) indicate statistical significance at *p* < 0.001. (E) Representative spatial mapping image from GSE175540. In this sample, co‐localization of hypoxic features, hepatic‐differentiated signature expression, and PROX1‐positive areas was observed. Across the 24 samples, PROX1 expression was generally scattered without consistent lymphatic structure delineation. Sample sizes: LVI+ (*n* = 11), LVI− (*n* = 20). Spatial transcriptomics data were generated using the 10× Visium platform (55 μm spot diameter, 100 μm center‐to‐center spacing). ALB = albumin; ALDOB = aldolase B; ccRCC = clear cell renal cell carcinoma; CTXN3 = cortexin 3; GSEA = gene set enrichment analysis; H‐RCC = hepatic‐differentiated renal cell carcinoma; HE = hematoxylin and eosin; HNF1A = hepatocyte nuclear factor 1A; HNF4A = hepatocyte nuclear factor 4A; HPD = 4‐hydroxyphenylpyruvate dioxygenase; LVI = lymphovascular invasion; NCCHE = National Cancer Center Hospital East; PROX1 = prospero homeobox 1; PT = proximal tubule; RCC = renal cell carcinoma; scRNA‐seq = single‐cell RNA sequencing; SLC22A8 = solute carrier family 22 member 8; UMAP = uniform manifold approximation and projection.

To explore the broader biological context of these single‐cell findings and their relevance to LVI, we examined the hepatic‐differentiated signature across spatial and bulk transcriptomic datasets. Pathway enrichment analysis of cluster 4 cells from GSE159115 showed upregulation of multiple metabolic pathways, including carbon metabolism, glycine/serine/threonine metabolism, and amino acid metabolism (Figure 5A, left panel). Additionally, liver‐specific gene sets were enriched (Figure 5A, right panel), supporting the hepatic‐differentiated phenotype of these cells.

We next developed a hepatic‐differentiated RCC gene signature using stringent filtering criteria from cluster 4, selecting genes with log_2_FC > 5 and adjusted *p* < 0.001, while excluding non‐functional genes (Table S5). When examining key representative genes from this signature in our NCCHE cohort, we observed higher expression in LVI+ tumors compared to LVI− tumors (Figure 5B). Furthermore, unsupervised hierarchical clustering using these signature genes suggested potential associations with LVI status and clinical outcomes (Figure 5C).

To explore the spatial context of these hepatic‐differentiated RCC cells, we analyzed public spatial transcriptomics data from ccRCC samples (GSE175540) [17]. Spatial clustering analysis divided the regions into tumor hypoxic, tumor normoxic, and non‐tumor domains across all 24 samples. The mean silhouette scores ranged from 0.32 to 0.47, indicating reasonable clustering quality (Figure S4). The relative proportion of these three regions varied across samples, with tumor hypoxic regions comprising 11%–45% of tissue spots in most samples (Figure S5). This analysis indicated that the hepatic‐differentiated RCC gene signature was expressed at significantly higher levels in hypoxic tumor regions compared to normoxic tumor and non‐tumor regions (*p* < 0.001, Figures 5D and S6). In a representative sample, co‐localization of hypoxic regions, hepatic‐differentiated signature expression, and PROX1‐positive areas was observed (Figure 5E). However, across the 24 samples, PROX1 expression was generally scattered without forming identifiable lymphatic structures, precluding systematic quantification.

## 4 Discussion

The TITANIA study enabled our exploratory multi‐omics profiling of ccRCC specimens with and without lymphovascular invasion. This analysis revealed significant differences in metabolic profiles, particularly the upregulation of ALDOB and other metabolic genes, in LVI+ specimens. Subsequent examination of public single‐cell RNA sequencing data uncovered a distinct hepatic‐differentiated ccRCC subpopulation expressing liver‐specific genes, including ALDOB. This subpopulation represented a terminal state in the differentiation trajectory from normal proximal tubule cells. Building on these findings, we developed a hepatic‐differentiated RCC gene signature, which was significantly enriched in LVI+ tumors from our cohort. Spatial transcriptomics analysis further demonstrated that cells expressing this signature predominantly localized to hypoxic tumor regions, suggesting a potential association between the hypoxic microenvironment, hepatic‐like differentiation, and lymphovascular invasion. The key findings of this study are summarized in the graphical abstract.

It is important to emphasize that our findings are preliminary observations from an exploratory study, lacking functional validation. Nevertheless, the identification of this hepatic‐differentiated subpopulation in ccRCC offers an intriguing hypothesis regarding metabolic reprogramming in cancer progression [25, 26]. Our results suggest that ccRCC cells may undergo paradoxical metabolic adaptation in hypoxic environments, adopting liver‐like metabolic programs to maintain energy‐intensive processes despite oxygen limitations [27]. This metabolic flexibility may confer a survival advantage, though this hypothesis requires functional validation.

ALDOB emerged as a central component of this hepatic‐differentiated signature. Its significant upregulation in LVI+ tumors suggests a potential role for altered fructose metabolism in ccRCC progression [28]. ALDOB catalyzes the reversible cleavage of fructose‐1,6‐bisphosphate, a key step in gluconeogenesis and fructose metabolism. ALDOB was prioritized as the central analytical focus of this study for three converging reasons: (1) it is the principal fructose‐metabolic enzyme that was consistently upregulated at both RNA and protein levels in LVI+ tumors; (2) it emerged as a major PPI hub node in proteomic network analysis; and (3) its expression was specifically concentrated in the hepatic‐differentiated subpopulation at single‐cell resolution, providing cross‐platform corroboration across all three analytical layers. Among other upregulated hepatic genes (HPD, DIO1, ANGPTL3), none exhibited this same convergence of evidence, nor the degree of PPI network centrality; they were therefore included in the gene signature (Table S5) rather than selected as primary anchoring candidates. In ccRCC, where the VHL‐HIF axis is constitutively activated, extensive metabolic rewiring occurs [29, 30]. The subpopulation‐specific ALDOB upregulation may represent a metabolic adaptation where enhanced fructose utilization provides an alternative energy source under hypoxic conditions. Unlike previous studies reporting variable ALDOB expression in ccRCC [31], our single‐cell analysis shows expression primarily confined to the hepatic‐differentiated subpopulation. This subpopulation‐specific pattern may explain inconsistent findings in prior bulk tissue studies and underscores the importance of single‐cell resolution for dissecting intratumoral metabolic heterogeneity. Our hepatocyte signature comparison confirmed that Cluster 4 selectively acquires metabolic programs while lacking non‐metabolic hepatocyte functions (Figures S2 and S3), indicating targeted metabolic adaptation rather than a complete lineage switch. This metabolic shift parallels findings by Bu et al. (2018), where colorectal cancer liver metastases upregulated ALDOB to enhance fructose metabolism [32]. While their study focused on metastatic adaptation to the liver, our findings suggest ccRCC cells undergo similar reprogramming within the primary tumor, particularly in hypoxic regions. This raises the possibility that the hepatic‐differentiated phenotype represents a pre‐metastatic adaptation, though this hypothesis requires functional validation.

### 4.1 Alternative Hypotheses for the Hepatic‐Differentiated Phenotype

An important question is whether Cluster 4 represents true trans‐differentiation toward a hepatic lineage or an alternative metabolic adaptation within the broader context of VHL‐HIF signaling. VHL loss in ccRCC leads to constitutive stabilization of HIF‐1α/HIF‐2α, driving extensive metabolic reprogramming including enhanced aerobic glycolysis, increased fatty acid synthesis, and suppression of the TCA cycle [29, 30]. While this VHL‐HIF–driven metabolic rewiring is a hallmark of ccRCC, it is noteworthy that the metabolic programs enriched in Cluster 4—gluconeogenesis, fructose metabolism, and apolipoprotein‐mediated lipid transport—do not correspond to the canonical HIF‐driven metabolic profile and instead overlap with liver‐specific metabolic functions. Lineage plasticity, whereby tumor cells acquire features of other cell types without genuine differentiation, has been described in various cancers [33] and may also occur in RCC [34]. HIF‐1α–mediated regulatory mechanisms [35] and non‐coding RNA networks [36] could further contribute to metabolic reprogramming in ccRCC. Our current data do not allow us to distinguish between these possibilities, and functional validation studies are essential to address this question. That said, we note that liver‐specific master transcription factors HNF1A and HNF4A [24, 37] were activated in Cluster 4, and that the overlap with hepatocyte signatures was restricted to metabolic functions while non‐metabolic hepatocyte programs were absent (Figure S3). These observations are at least consistent with a degree of transcriptional reprogramming beyond what would be expected from HIF‐driven metabolic shifts alone, although alternative explanations cannot be excluded.

The transcriptional regulation of this phenotype appears to involve liver‐specific transcription factors, HNF1A and HNF4A [37], which were significantly activated in the hepatic‐differentiated ccRCC subpopulation [24, 37]. Their role in driving liver‐specific gene expression programs in a kidney tumor highlights striking cellular plasticity and transcriptional reprogramming during tumor evolution. The spatial association between hepatic‐differentiated signature expression and hypoxic regions provides supportive evidence for a potential link with lymphovascular invasion. However, PROX1 expression across the 24 samples was generally scattered, and systematic co‐localization quantification was not feasible.

The TITANIA framework provided standardized tissue collection with minimal cold ischemia time (median 12 min). Analysis of data quality confirmed no significant correlation between cold ischemia time and proteomic missing value rates (Figure S1), supporting consistent molecular integrity across all samples.

### 4.2 External Validation in TCGA‐KIRC

To assess clinical relevance in an independent cohort, we analyzed TCGA‐KIRC RNA‐seq data [1, 20, 21]. Among patients who underwent curative‐intent surgery and for whom DFI data were available (*n* = 115), the high signature score group showed a shorter disease‐free interval (log‐rank *p* = 0.009; Figure S7). However, this result should be interpreted with caution, as the optimal cutpoint method may overestimate statistical significance and bulk RNA‐seq captures the whole‐tumor average, potentially diluting a subpopulation‐level signal identified at single‐cell resolution. Specifically, results obtained with post hoc optimal thresholding should not be interpreted as confirmatory of the gene signature's prognostic value, and independent prospective validation with a pre‐specified cutpoint is warranted. This underscores the necessity of single‐cell approaches for detecting biologically meaningful but quantitatively minor tumor subpopulations.

Beyond its role as a potential prognostic indicator, the hepatic‐differentiated signature may carry broader therapeutic implications. Tumors expressing this phenotype show upregulation of ALDOB and related hepatic metabolic enzymes that are not typically targeted by standard anti‐angiogenic or immune checkpoint therapies currently used in ccRCC management. Should functional validation confirm that this subpopulation contributes to aggressive behavior, targeting the HNF1A/HNF4A transcriptional axis or downstream metabolic enzymes may represent a complementary therapeutic strategy. Furthermore, the distinctive molecular phenotype—characterized by liver‐specific surface proteins and metabolic programs—may open possibilities for imaging‐based detection of this subpopulation. Prior work has demonstrated that expression of specific membrane‐associated proteins can be visualized by MRI at the cellular level [38], and that tumor‐specific biomarkers such as PD‐L1 can be detected by near‐infrared fluorescence/Cerenkov luminescence dual‐modality imaging [39]; analogous approaches targeting hepatic‐differentiated tumor cells may be feasible but require dedicated future exploration.

Our study has several important limitations. First, the sample size is small (*n* = 31) with only 2 progression events, precluding multivariate analysis. Second, this study lacks functional validation; the associations reported are correlative and hypothesis‐generating. In vitro studies (e.g., ALDOB/HNF1A/HNF4A knockdown in ccRCC cell lines) and in vivo models are identified as essential next steps and are described as immediate priorities for our ongoing research program. Third, the public scRNA‐seq dataset (GSE159115) lacks lymphovascular invasion status and detailed pathological annotations, precluding direct correlation between Cluster 4 abundance and LVI. Additionally, the scRNA‐seq re‐analysis was performed without formal inter‐sample batch correction; while the biological validity of Cluster 4 was confirmed by three independent downstream approaches (hepatocyte signature comparison, module score analysis, and spatial transcriptomics), a contribution of donor‐level technical variation to cluster definition cannot be fully excluded. Fourth, TCGA‐KIRC bulk RNA‐seq data has inherent limitations for validating a subpopulation‐level phenotype, as the signal is diluted in whole‐tumor averages. Additionally, the use of an optimally selected cutpoint for dichotomization may inflate statistical significance, and independent validation with a pre‐specified threshold is warranted. Fifth, PROX1 co‐localization with hypoxic regions was observed in one representative sample only; across 24 samples, PROX1 expression was generally scattered. Sixth, alternative pseudotime trajectories were not explored. Seventh, the higher TMB in LVI+ tumors may be confounded by the significantly older age of these patients. Finally, the short follow‐up period (median 18.3 months) limits prognostic assessment.

Despite these limitations, our exploratory findings provide a foundation for more targeted investigations. In conclusion, our multi‐omics analysis identified a unique hepatic‐differentiated subpopulation in ccRCC associated with lymphovascular invasion. This discovery‐based study offers a hypothesis that this metabolic phenotype may contribute to aggressive disease, warranting future functional validation.

## Author Contributions

Conceptualization: S.Y., Y.T., T.Y., M.I. Data curation: R.Y., T.F., N.G., H.J., Y.K. Formal analysis: S.Y., R.Y., T.F. Investigation: S.Y., Y.T., T.K., G.I., R.W. Methodology: R.Y., T.F., N.G., H.J. Project administration: Y.T., T.Y., Y.K., M.I. Resources: Y.T., T.Y., M.I., H.M., N.G., H.J., R.W. Software: R.Y., T.F. Supervision: Y.T., T.Y., M.I., H.M. Validation: S.Y., R.Y., T.F. Visualization: S.Y., R.Y., T.F. Writing – original draft: S.Y. Writing – review and editing: All authors.

## Funding

The authors have nothing to report.

## Ethics Statement

The study was approved by the National Cancer Center Hospital East Institutional Review Board (IRB number: 2020‐079). All patients provided written informed consent. The study was performed in accordance with the Declaration of Helsinki.

## Conflicts of Interest

Takayuki Yoshino reports honoraria from Chugai Pharmaceutical, Takeda Pharmaceutical, Merck Biopharma, Bayer Yakuhin, Ono Pharmaceutical and MSD K.K; consulting fee from Sumitomo Corp; research funding from Amgen K.K., Bristol‐Myers Squibb K.K., Caris MPI Inc., Chugai Pharmaceutical Co. Ltd., Daiichi Sankyo Co. Ltd., Eisai Co. Ltd., Exact Sciences Corporation, FALCO biosystems Ltd., GlaxoSmithKline K.K., IQVIA Services Japan K.K., Medical & Biological Laboratories Co. Ltd., Merus N.V., Miyarisan Pharmaceutical Co. Ltd., Molecular Health GmbH, MSD K.K., Natera Inc., Nippon Boehringer Ingelheim Co. Ltd., Ono Pharmaceutical Co. Ltd., Pfizer Japan Inc., Roche Diagnostics K.K., Sanofi K.K., Sysmex Corp., Taiho Pharmaceutical Co. Ltd. and Takeda Pharmaceutical Co. Ltd. Nina Gabelia and Hartmut Juhl are employees of Indivumed GmbH, which generated and provided the multi‐omics data used in this study. Other authors have no conflicts of interest to declare.

## Supporting information


**Figure S1:** Cold ischemia time versus proteomic data quality. Scatter plots: (A) whole proteome (*ρ* = 0.11, *p* = 0.555), (B) phospho‐peptides (*ρ* = 0.168, *p* = 0.367), (C) phospho‐sites (*ρ* = 0.15, *p* = 0.42). No significant correlations observed.

**Figure S2:** Hepatocyte module score analysis. Violin plots showing hepatocyte signature module scores across all clusters for three independent databases: (A) CellMarker 2.0, (B) Human Protein Atlas, (C) MacParland et al. [19]. Cluster 4 shows the highest scores (Wilcoxon *p* < 0.001).

**Figure S3:** Hepatocyte functional category analysis. Bar plot showing percentage of hepatocyte category genes found among Cluster 4 DEGs: gluconeogenesis (3/5, 60%), lipid transport (5/11, 45%), amino acid metabolism (4/10, 40%), CYP450 (2/12, 17%), coagulation (0/10, 0%), transporters (0/6, 0%), one‐carbon (0/3, 0%).

**Figure S4:** Clustering quality assessment across 24 spatial transcriptomics samples from GSE175540.

**Figure S5:** Region distribution across all spatial transcriptomics samples, displaying the proportion of tumor hypoxic, tumor normoxic, and non‐tumor regions.

**Figure S6:** Hepatic‐differentiated RCC gene signature score distribution by region type across individual spatial transcriptomics samples.

**Figure S7:** External validation using TCGA‐KIRC cohort. Kaplan–Meier curve for Disease‐Free Interval (DFI) in TCGA‐KIRC patients who underwent curative‐intent surgery and for whom DFI data were available (*n* = 115; Low *n* = 88, High *n* = 27). Log‐rank *p* = 0.009. Cutpoint determined by maximally selected rank statistics (surv_cutpoint).

**Table S1:** Whole‐genome sequencing coverage statistics for all 31 ccRCC samples.

**Table S2:** Comparison of tumor mutational burden (TMB) and chromosomal instability metrics (CIN‐FGA, CIN‐CNA, CIN‐CNH) between LVI+ (*n* = 11) and LVI− (*n* = 20) groups. TMB: LVI+ median 3.37 vs. LVI− 2.31 mutations/Mb, Wilcoxon *p* = 0.030.

**Table S3:** CNV distribution of 15 key genes stratified by LVI status. Fisher's exact tests: no significant differences for any gene.

**Table S4:** Detailed characterization of all 14 cell clusters identified in single‐cell RNA sequencing analysis of GSE159115.

**Table S5:** Complete list of 105 genes comprising the hepatic‐differentiated RCC gene signature with their expression statistics.

## Data Availability

The multi‐omics data from the NCCHE cohort were generated as part of the international TITANIA collaborative project. Public deposition of these data was planned but is currently suspended due to patient privacy considerations under institutional and consortium policies. Processed data supporting the findings are available from the corresponding author upon reasonable request, subject to institutional approval. Public datasets used in this study are available through the Gene Expression Omnibus (GSE159115, GSE175540). TCGA‐KIRC data are publicly available through the GDC portal (https://portal.gdc.cancer.gov/) and UCSC Xena (https://xenabrowser.net/).
