# Supplementary figures and images for: Hepatic‐Differentiated Subpopulation in Clear Cell Renal Cell Carcinoma: A Multi‐Omics Analysis of Tumors With Lymphovascular Invasion
Source: Cancer Med. 2026 Apr 14;15(4):e71843. doi: 10.1002/cam4.71843 (PMC13079427; doi:10.1002/cam4.71843)

**A****Whole proteome**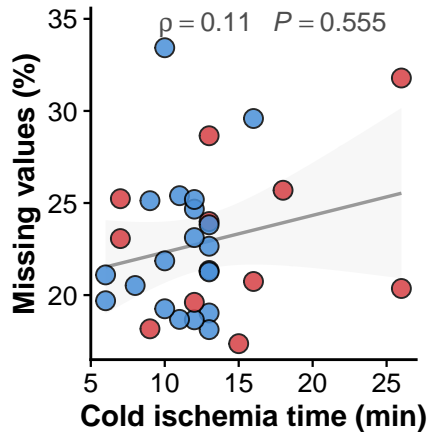**B****Phospho-peptides**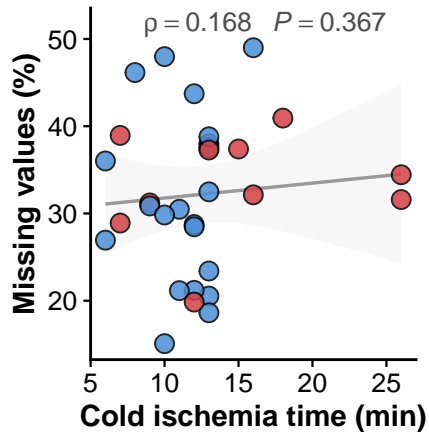**C****Phospho-sites**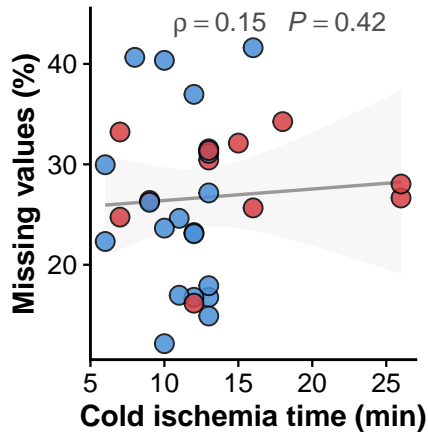

Supplement: Supplementary file 1 — Figure S1: Cold ischemia time versus proteomic data quality. Scatter plots: (A) whole proteome (ρ = 0.11, p = 0.555), (B) phospho‐peptides (ρ = 0.168, p = 0.367), (C) phospho‐sites (ρ = 0.15, p = 0.42). No significant correlations observed. [file CAM4-15-e71843-s001.pdf]

CellMarker 2.0

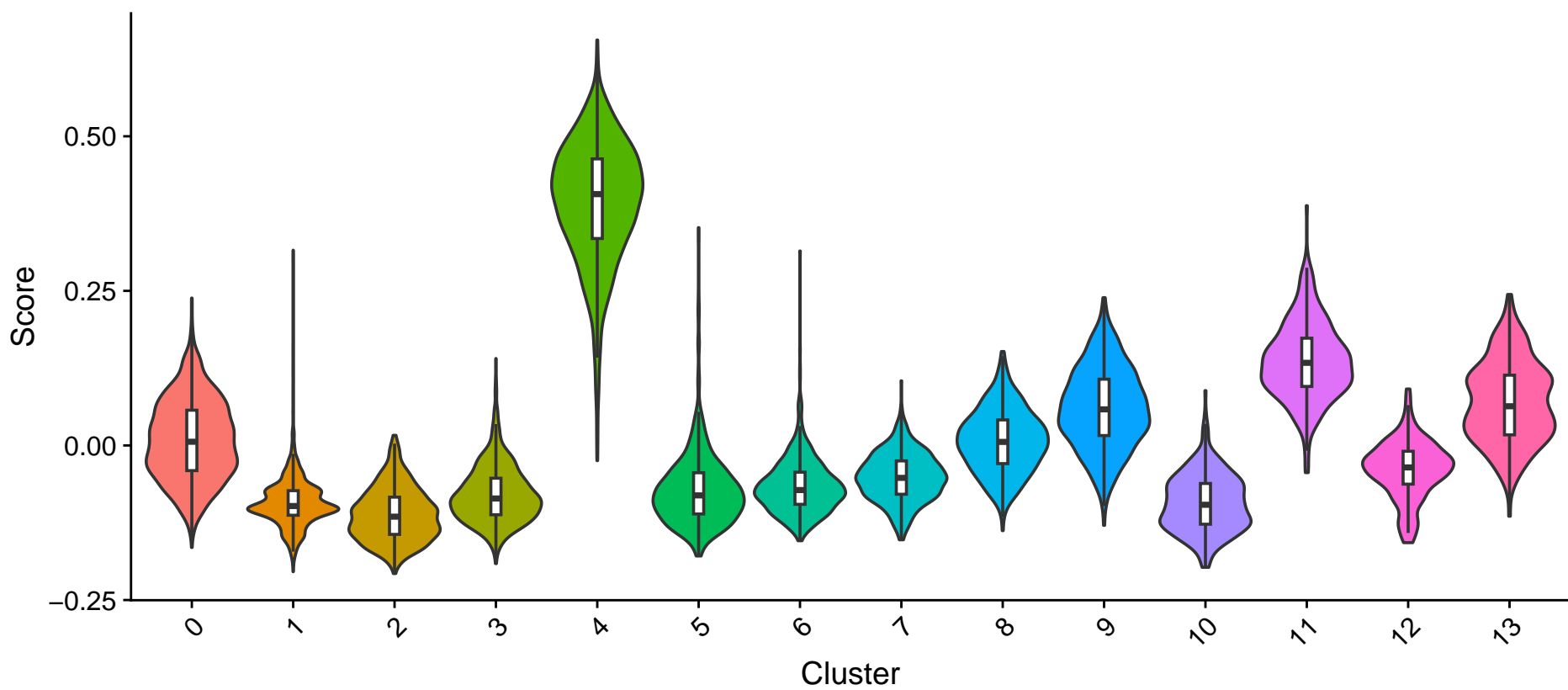

HPA Liver-Enriched

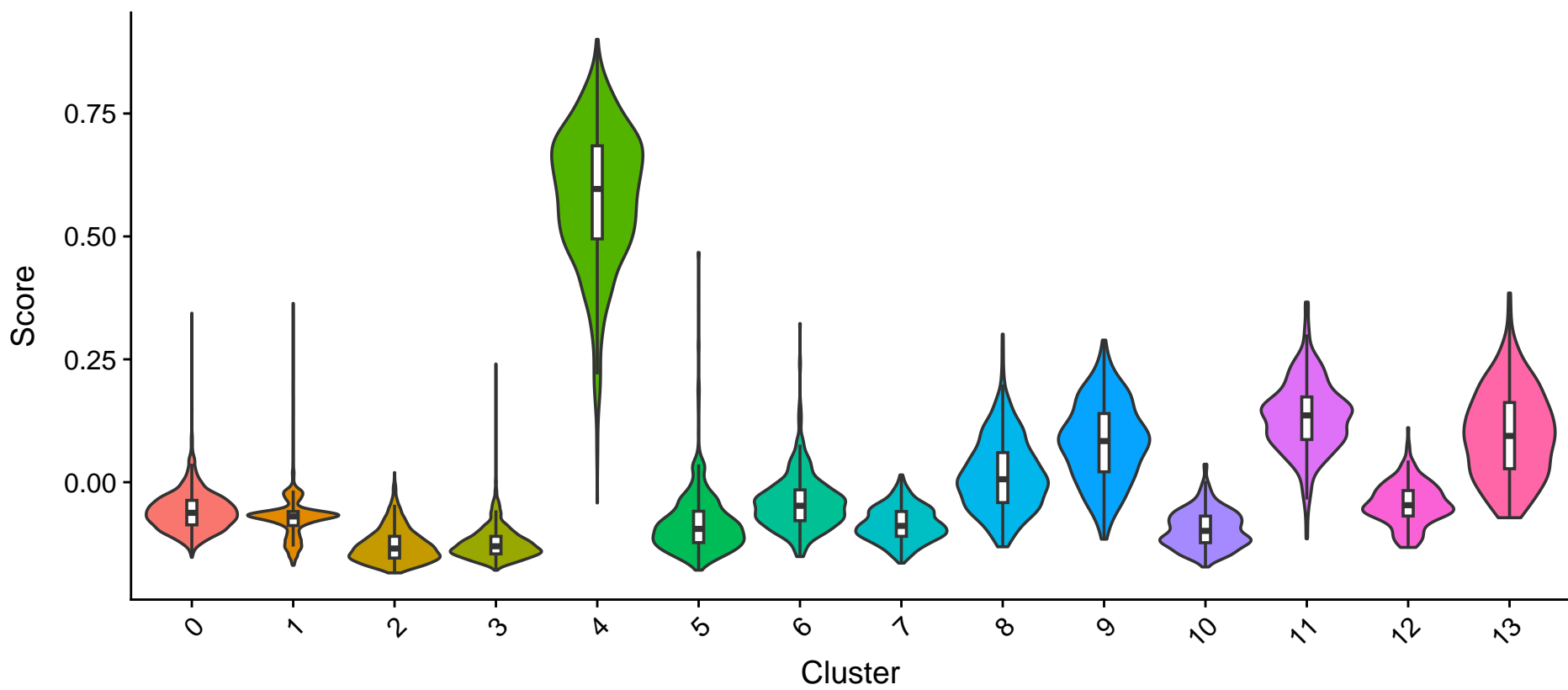

MacParland et al. 2018

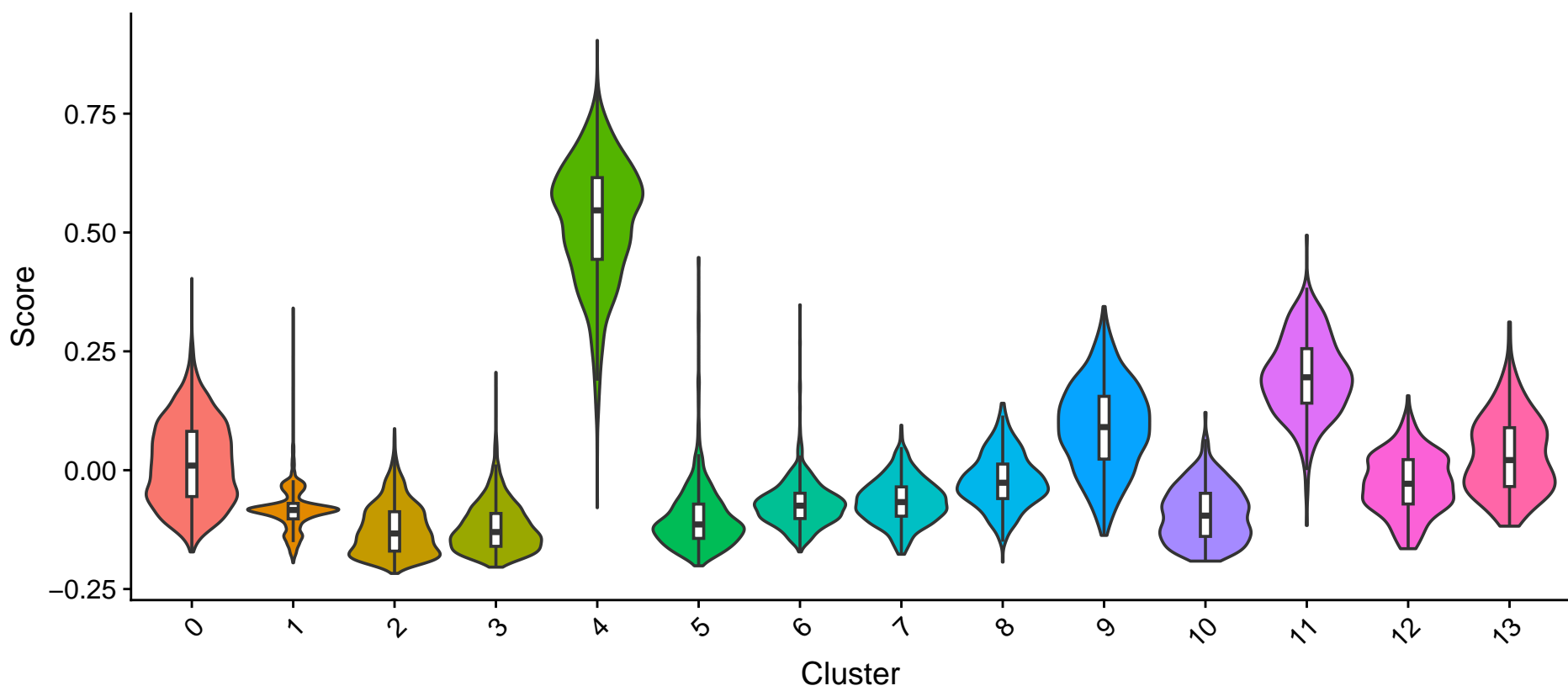

Supplement: Supplementary file 2 — Figure S2: Hepatocyte module score analysis. Violin plots showing hepatocyte signature module scores across all clusters for three independent databases: (A) CellMarker 2.0, (B) Human Protein Atlas, (C) MacParland et al. [19]. Cluster 4 shows the highest scores (Wilcoxon p < 0.001). [file CAM4-15-e71843-s003.pdf]

## Hepatocyte Functional Pathways Represented in Cluster 4

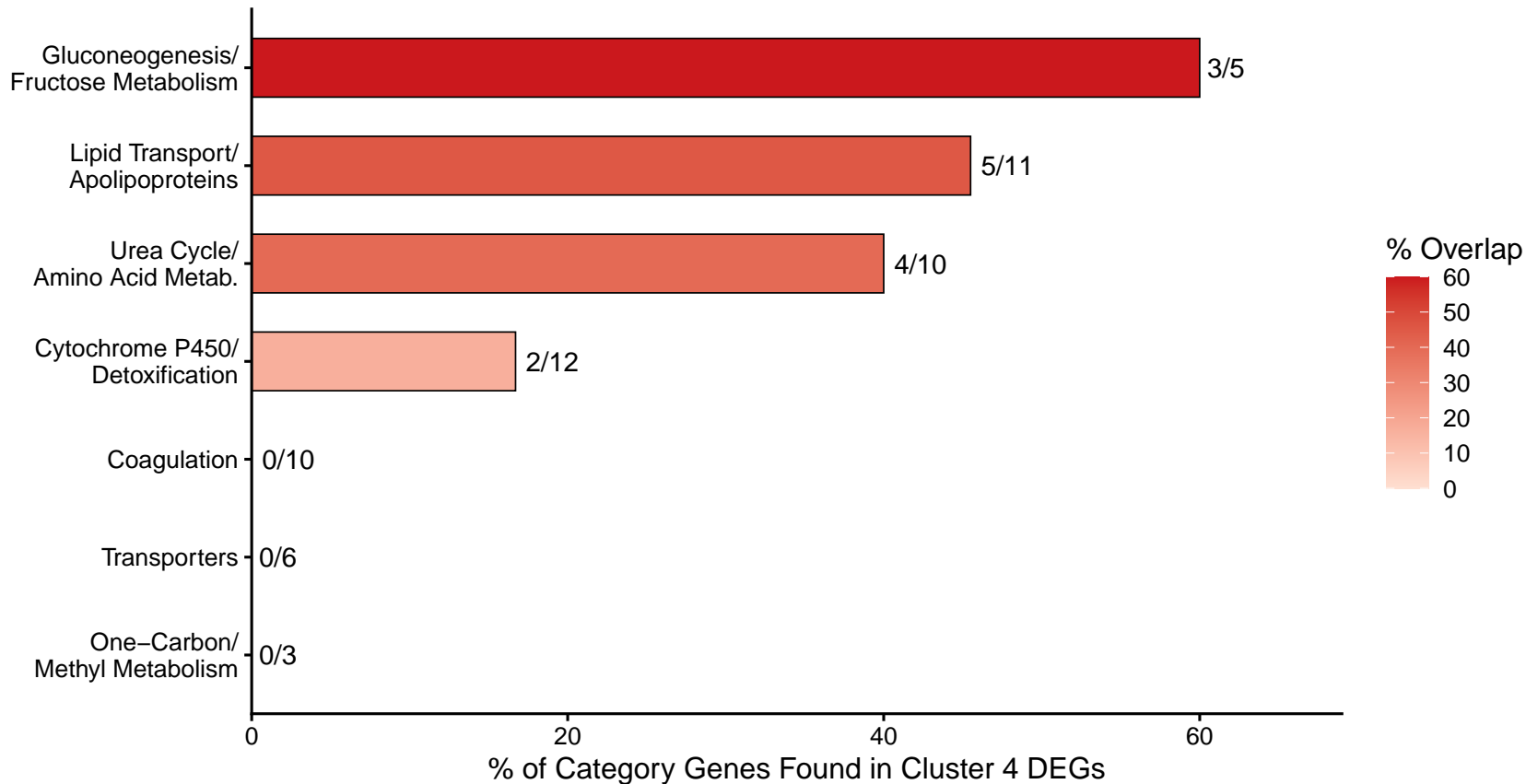

Supplement: Supplementary file 3 — Figure S3: Hepatocyte functional category analysis. Bar plot showing percentage of hepatocyte category genes found among Cluster 4 DEGs: gluconeogenesis (3/5, 60%), lipid transport (5/11, 45%), amino acid metabolism (4/10, 40%), CYP450 (2/12, 17%), coagulation (0/10, 0%), transporters (0/6, 0%), one‐carbon (0/3, 0%). [file CAM4-15-e71843-s009.pdf]

Clustering Quality Across Samples

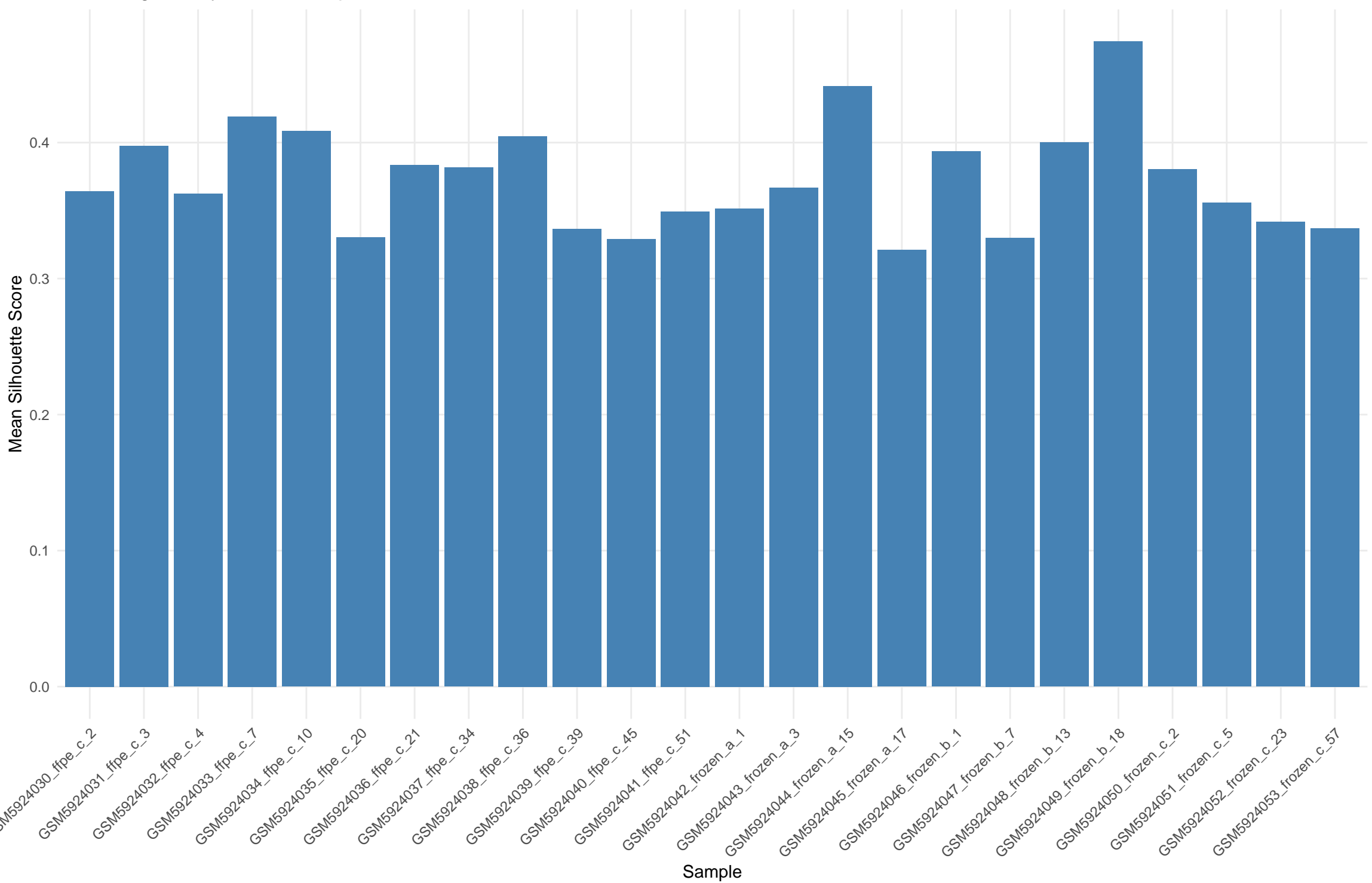

Supplement: Supplementary file 4 — Figure S4: Clustering quality assessment across 24 spatial transcriptomics samples from GSE175540. [file CAM4-15-e71843-s007.pdf]

# Region Distribution Across Samples

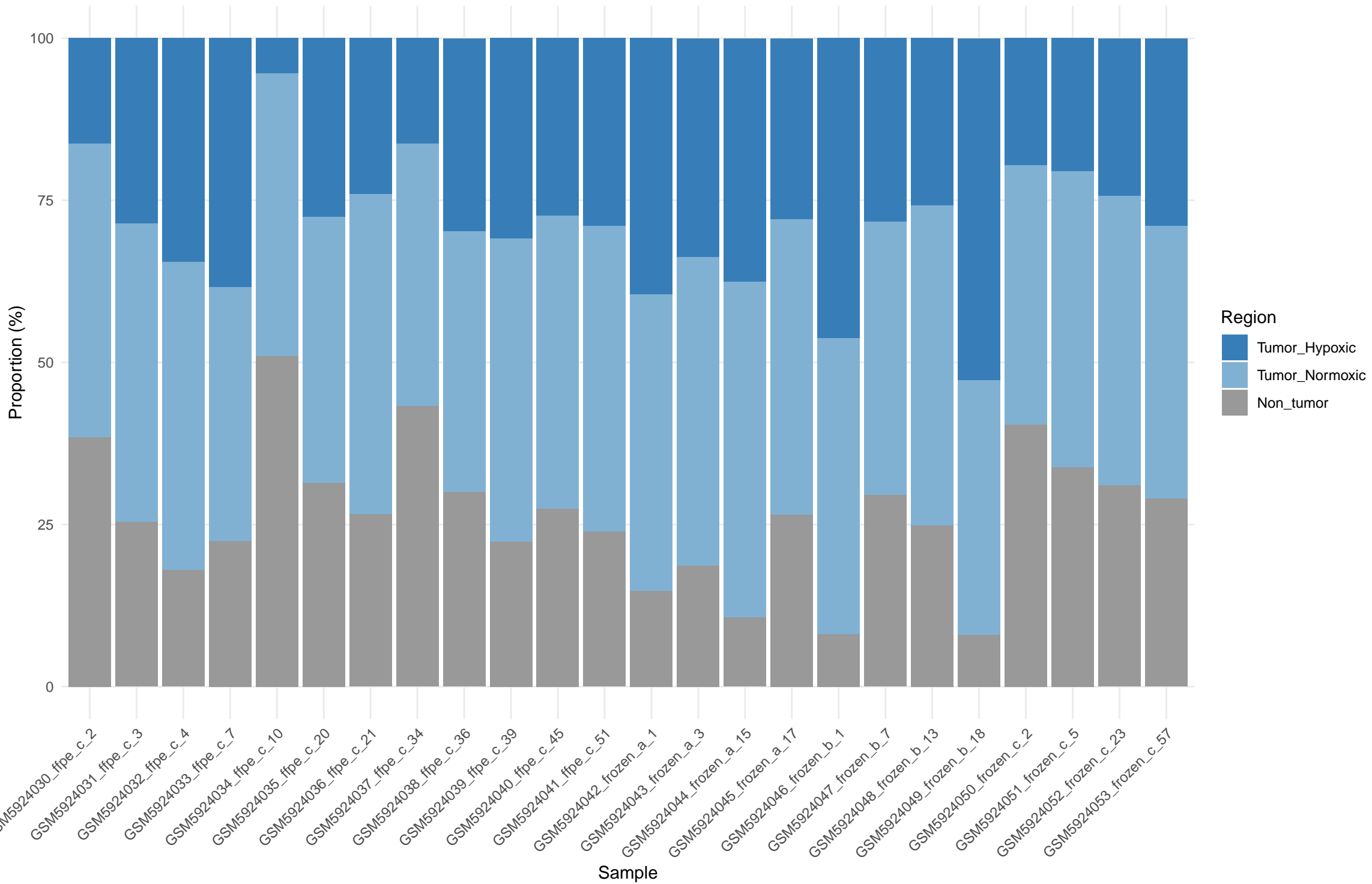

Supplement: Supplementary file 5 — Figure S5: Region distribution across all spatial transcriptomics samples, displaying the proportion of tumor hypoxic, tumor normoxic, and non‐tumor regions. [file CAM4-15-e71843-s005.pdf]

### Hepatic-Differentiated RCC Score Distribution by Sample

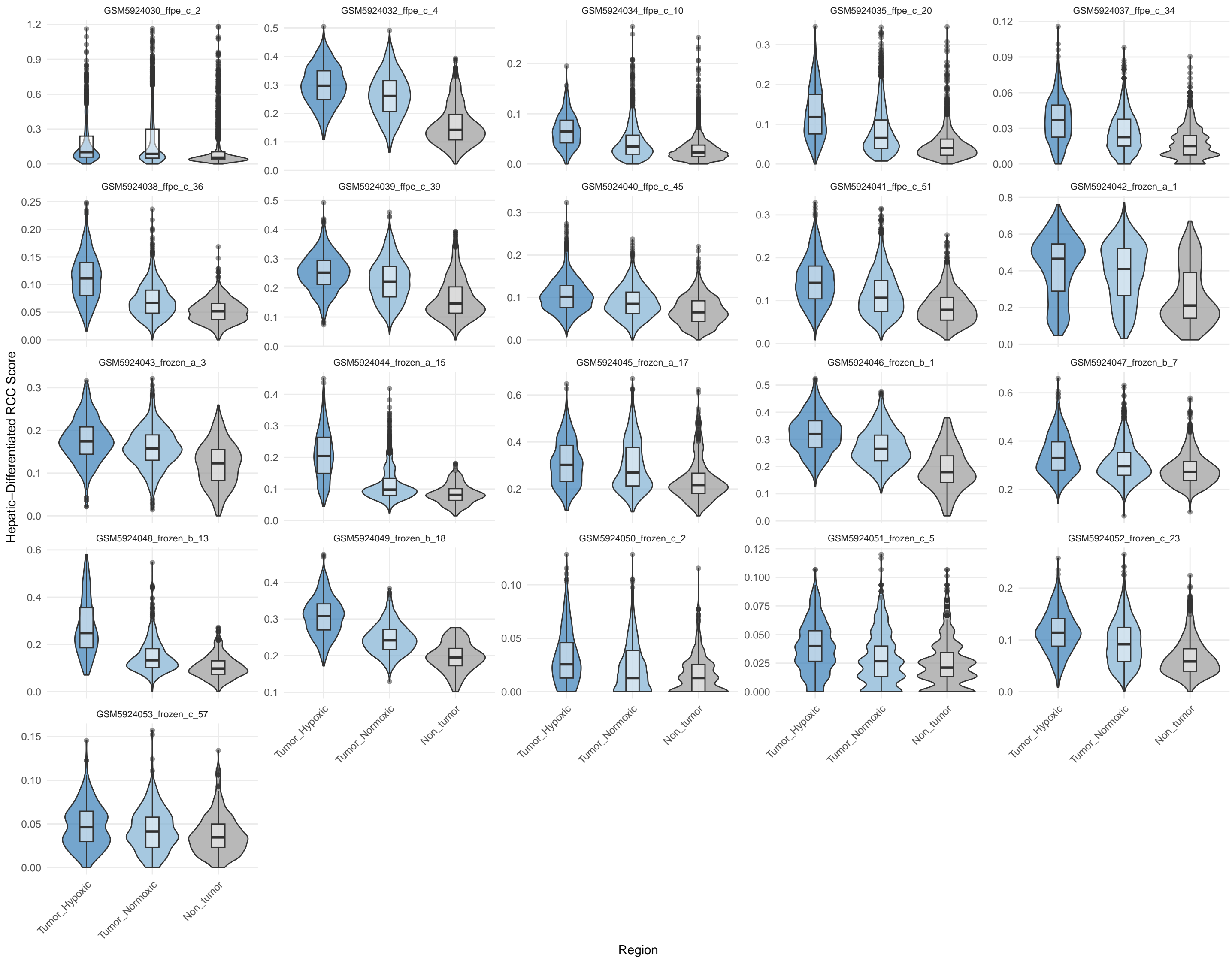

Supplement: Supplementary file 6 — Figure S6: Hepatic‐differentiated RCC gene signature score distribution by region type across individual spatial transcriptomics samples. [file CAM4-15-e71843-s012.pdf]
