# Supplementary material for: Hepatic‐Differentiated Subpopulation in Clear Cell Renal Cell Carcinoma: A Multi‐Omics Analysis of Tumors With Lymphovascular Invasion
Source: Cancer Med. 2026 Apr 14;15(4):e71843. doi: 10.1002/cam4.71843 (PMC13079427; doi:10.1002/cam4.71843)
Supplement: Supplementary file 7 — Figure S7: External validation using TCGA‐KIRC cohort. Kaplan–Meier curve for Disease‐Free Interval (DFI) in TCGA‐KIRC patients who underwent curative‐intent surgery and for whom DFI data were available (n = 115; Low n = 88, High n = 27). Log‐rank p = 0.009. Cutpoint determined by maximally selected rank statistics (surv_cutpoint). [file CAM4-15-e71843-s011.pdf]

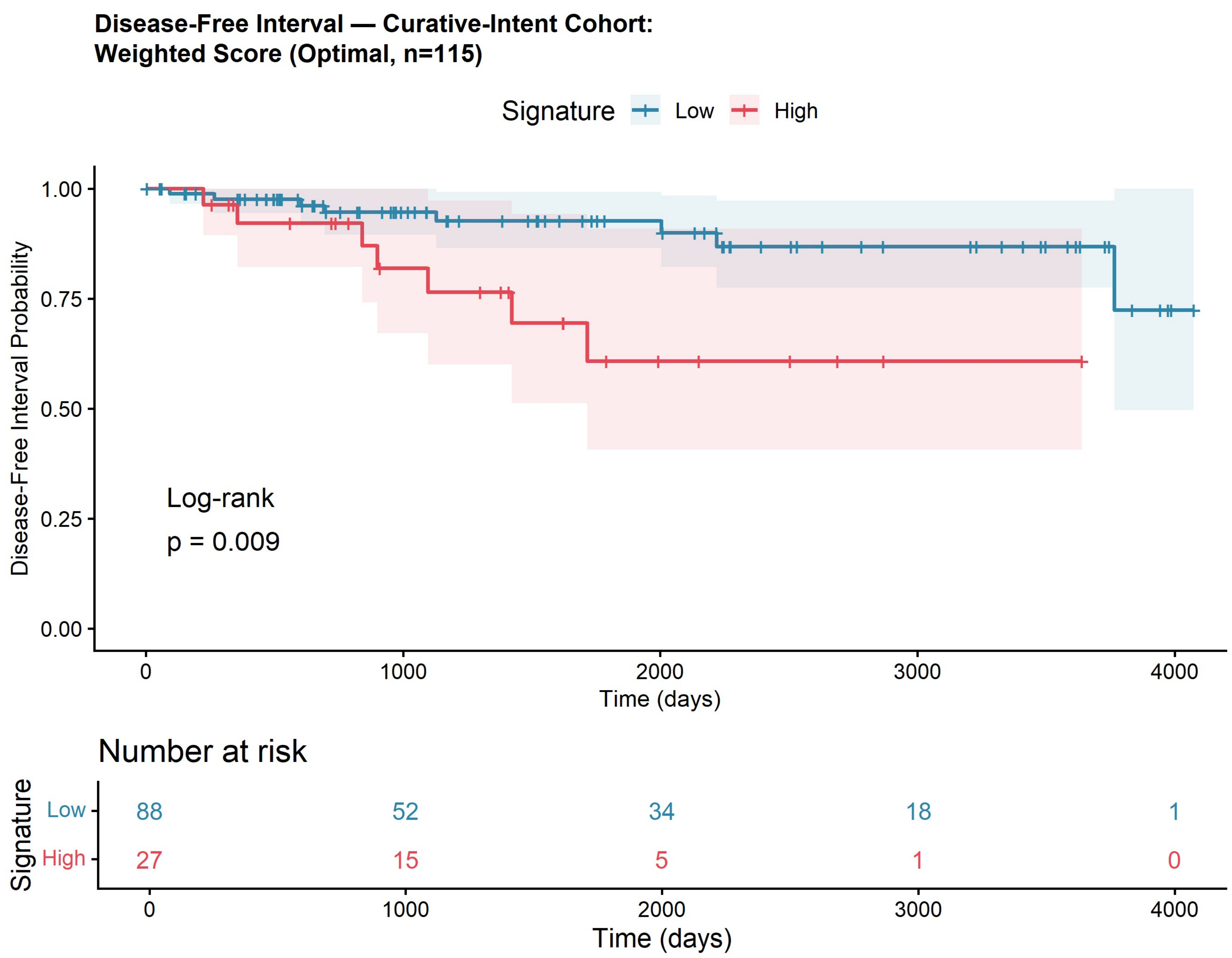

Disease-Free Interval — Curative-Intent Cohort:
Weighted Score (Optimal, n=115)
Signature
Low
High
1.00
0.75
0.50
0.25
0.00
Disease-Free Interval Probability
Log-rank
p = 0.009
0
1000
2000
3000
4000
Time (days)
Number at risk
Signature
Low
High
88
52
34
18
1
27
15
5
1
0
0
1000
2000
3000
4000
Time (days)
